# Assessment of Psychological Distress in Surgical Patients: A Comparison Between Day Care and Long-Term Hospitalization in an Oncology Hospital

**DOI:** 10.3390/healthcare14050626

**Published:** 2026-03-02

**Authors:** Maria Kapritsou, Theodoros N. Sergentanis, Nikolaos Maniadakis, Vasiliki Papanikolaou

**Affiliations:** 1 Oncological Sector, General Oncology Hospital of Athens ‘’Saint Savvas’’, 11522 Athens, Greece; 2 Department of Public Health Policy, University of West Attica, 11521 Athens, Greece

**Keywords:** elective surgery, preoperative waiting time, stress, preoperative depression, oncology hospital, health management

## Abstract

**Introduction:** Patients undergoing surgery are exposed to various stressors that may increase psychological distress during the perioperative period. These repercussions may be substantial, affecting both physical and mental health, as well as the ability to resume regular activities and overall quality of life. Aim: This study aimed to compare the preoperative psychological distress levels of patients admitted to long-term preoperative hospitalization (LONG) to those of patients admitted to day care (DC) facilities for ambulatory surgery within an oncology hospital and to examine potential sociodemographic predictors. **Methods:** This was a prospective observational study that included 176 individuals who underwent surgery in two cohorts. Patients in the DC cohort (*n* = 88) were treated in a day care surgery clinic, whereas patients in the LONG cohort (*n* = 88) were treated in a long-term oncology hospital. Demographic and clinical data were collected. Patients’ psychological distress (depression, anxiety, and stress) was preoperatively evaluated using the DASS-42. Univariate and multivariate logistic regression analyses were performed. **Results:** The DASS-42 scale’s Cronbach’s alpha was 0.923. There was no significant difference between the cohorts in terms of age; however, waiting time before surgery differed significantly (U = 2884, *p* = 0.002). Stress levels differed significantly between the two cohorts (*p* = 0.05). **Conclusions:** Health managers and health care providers should consider gender, surgical severity, and rural/urban residence as factors associated with preoperative psychological distress. Studies assessing gender-specific dynamics, as well as mixed-methods approaches, could provide deeper insights into patients’ experiences and the correlations of distress and highlight implications for oncology nursing practice across different hospitalization models.

## 1. Introduction

The demand for elective surgery is increasing significantly due to technological innovations and increasing patient expectations. Hospital managers face difficulties in balancing supply and demand, often confronting long waiting lists that generate public concern and raise issues of equity [1]. This is especially true for elective procedures, which are chosen by patients in consultation with their doctors for better quality of life. Prolonged waiting time (WT) for these services may influence patients’ decisions to seek private providers or forgo treatment [2].

According to the report “Unmet Health Care Need Statistics” [3] in Italy, 29.9% of the population aged over 15 years in need of health care reported foregoing treatment due to long waiting lists; thus, WT is carefully considered by policymakers. WT is an indicator integrating patient satisfaction, efficiency, and horizontal equity in health care delivery and economic status; preoperative psychiatric consultation has been suggested to increase patients’ emotional readiness [4]. Solutions aiming to reduce WT involve strengthening supply, increasing the availability of resources (opening hours, staff and beds), staffing, or paying extra financial support to health care professionals to increase productivity. However, those solutions have not been able to radically reduce the system-wide WTs [4].

The examination of stress, anxiety, and depression levels among surgical patients in relation to their residential backgrounds has demonstrated a significant evolution of understanding within the field. Early studies focused predominantly on the psychological impact of physical illnesses, where stress and anxiety were viewed primarily as transient responses to surgery, largely disconnected from external factors such as living environment [5,6]. Nevertheless, the intersection between mental health and sociodemographic variables highlights the need for a nuanced understanding of how these factors shape the patient experience in outpatient surgical settings [7].

The aim of the present study was not to compare clinically identical groups, but to explore differences in preoperative psychological distress between two distinct hospitalization pathways within the same oncology hospital: day-care (DC) surgery and long-term preoperative hospitalization (LONG). These pathways inherently reflect different clinical and organizational realities, which form part of the real-world context of oncological surgical care.

## 2. Methods

### 2.1. Study Design and Sample

The sample of this prospective observational study (June 2020 to June 2021) consisted of patients who underwent surgery (*n* = 176). Eighty-eight patients were recruited from the day care center “N. Kourkoulos”, Oncological Hospital “Saint Savvas” (*n* = 88, DC cohort), and 88 patients from the Oncological Hospital “Saint Savvas” (*n* = 88, LONG cohort). Based on a power analysis conducted using G*Power software version 3.1.9.7 (University of Duesseldorf, Germany) at statistical significance α = 0.05 and an effect size of d = 0.43, the sample of 176 patients was adequate for the achievement of 0.80 statistical power in two-tailed tests. The sample size calculation was based on anticipated differences in mean DASS-42 scores between groups. Although subsequent regression analyses used dichotomized outcomes, the original power estimation was conducted for continuous comparisons, which should be considered when interpreting statistical precision.

Allocation to the DC or LONG cohort was determined by the hospital’s established clinical pathways and surgical planning procedures. In general, patients undergoing major oncological surgeries requiring postoperative monitoring were admitted to the LONG unit, whereas patients scheduled for mild or moderate procedures considered clinically appropriate for same-day discharge were treated in the day care center. The research team did not influence patient allocation.

### 2.2. Data Collection and Assessment of Psychological Distress Levels

The following information was collected: demographic information (gender, age, education, marital status, place of residence, professional status), the severity grading of the surgery, whether the operation was the first or not, and waiting time (WT). 

Psychological distress was assessed using the DASS-42 scale, which measures negative emotional states (Depression, Anxiety, Stress) with a set of three self-administered scales consisting of 42 items. Each of the three DASS subscales includes 14 items; participants indicate the degree to which they consider each of the sentences to represent them using a 4-point Likert scale (0 = not applicable to me at all, 3 = applied to me too much, or most of the time) [8].

### 2.3. Ethical Issues

The research protocol was approved by the hospital’s Scientific Committee (ID: 19749/19 June 2020). Patients signed informed consent forms for their participation in the study. In the event that the patient declined to take part in the study, neither the doctors nor the nurses were informed, and subsequent care was unaffected. Additionally, the study protected the confidentiality and anonymity of the data.

### 2.4. Statistical Analysis

The statistical analysis was carried out using the statistical software SPSS 25.0 (IBM Corp., Armonk, NY, USA). Descriptive data were analyzed, and mean, median, and standard deviation values were calculated. Continuous variables were assessed for normality. If normally distributed, a *t*-test was used to compare the DC versus the LONG cohort; on the other hand, the Mann–Whitney U test was used for comparisons in cases of non-normal distribution.

The primary outcome of the study was preoperative psychological distress as measured by the DASS-42 total score. For regression analyses, the total DASS-42 score was categorized into “normal” versus “mild/moderate/severe/very severe” according to established DASS-42 severity thresholds. This dichotomous variable was used as the dependent variable in logistic regression models. Subscale-specific analyses (depression, anxiety, stress) followed the respective validated cut-offs of the DASS-42.

Values of Cronbach’s alpha > 0.7 were considered acceptable for the DASS-42 overall questionnaire and scales. Univariate and multivariate binary logistic regression analyses were conducted, with the dependent variable being participants’ mental health status as evaluated by the DASS-42, and independent variables gender (female vs. male), age (≥median vs. <median), education (secondary/university vs. primary/none), marital status (married vs. single/divorced/widowed), place of residence (urban vs. rural/semiurban), professional status (in work/studying vs. unemployed/household/retired), WT (≥median vs. <median), severity of surgery (major vs. mild; moderate vs. mild), first operation (yes vs. no).

Separate analyses were undertaken in the two cohorts (LONG and DC cohorts), as they represent different health system structures and approaches. The level of statistical significance was set at 0.05.

## 3. Results

Initially, 183 patients were approached for the study, of whom 176 agreed to participate, yielding a response rate of 96.2%. Table 1 presents the demographic and clinical data of the patients in the DC (*n* = 88) and LONG cohorts (*n* = 88).

Regarding the reliability of the DASS-42 questionnaire, Cronbach’s alpha = 0.923 for the total score. The Cronbach’s alpha values for the DASS-42 scales were 0.902, 0.904, and 0.829 for the stress, depression, and anxiety scales, respectively.

### 3.1. Interactions in the LONG Cohort

In the LONG cohort, gender could not be introduced in the logistic regression models, as all males (26/26) scored below the DASS-42 threshold for psychological distress versus 41/62 females (*p* < 0.001, Fisher’s exact); the respective pattern arose regarding the DASS-42 depression scale (26/26 males vs. 45/62 females scoring below 9, *p* = 0.002) and the DASS-42 stress scale (26/26 males vs. 42/62 females scoring below 14, *p* < 0.001).

The overall DASS-42 scores for depression-anxiety-stress levels were associated with place of residence and severity of surgery. Specifically, residents of urban areas had 3.11 times higher odds of psychological distress (95% CI: 1.02–9.45, *p* = 0.046) than rural/semiurban residents. Patients undergoing major surgery reported lower levels of psychological distress (unadjusted OR = 0.04, 95% CI: 0.004–0.44, *p* = 0.008) versus those undergoing mild surgery (Table 2). At the multivariate analyses, the aforementioned associations remained statistically significant; however, these estimates should be interpreted with caution given the sample size and model instability (adjusted OR = 4.66, 95% CI: 1.22–17.81, *p* = 0.024 for the urban vs. rural/semiurban comparison; adjusted OR = 0.02, 95% CI: 0.002–0.3, *p* = 0.004, for the major vs. mild comparison), whereas also moderate surgery emerged with lower psychological distress levels versus mild surgery (adjusted OR = 0.06, 95% CI: 0.01–0.76, *p* = 0.030).

In Table 3, the association between the DASS-42 scales and the sociodemographic and clinical variables is presented. In the unadjusted model, the depression levels were inversely associated with major and moderate severity of surgery vs. mild (*p* = 0.002 and *p* = 0.049, respectively). No multivariate analysis was conducted, as surgical severity was the only variable significantly associated with depression levels.

Regarding anxiety, both in the univariate and multivariate analysis, female gender (adjusted OR = 20.78, 95% CI: 2.10–205.38, *p* = 0.009) was associated with higher odds of anxiety; however, the wide confidence interval indicates limited precision of the estimate.

Concerning stress, both in the univariate and the multivariate analysis, urban residents (adjusted OR = 7.19, 95% CI: 1.52–34.07, *p* = 0.013) presented with higher odds of stress, whereas an inverse association was observed with major and moderate surgical severity; however, given the very low odds ratios and wide confidence intervals, these findings should be interpreted cautiously (major surgery: adjusted OR = 0.02, 95% CI: 0.001–0.26, *p* = 0.003; moderate surgery: adjusted OR = 0.04, 95% CI: 0.003–0.55, *p* = 0.017).

### 3.2. Correlations in the DC Cohort

Commenting on the overall DASS-42 scores, residents of urban areas presented with lower psychological distress levels than those residing in rural/semiurban areas (unadjusted OR = 0.16, 95% CI: 0.05–0.50, *p* = 0.002). No multivariate analysis was presented, as the rural/urban status was the only variable associated with overall psychological distress levels.

With respect to depression, both at the univariate and multivariate analysis, females presented with lower odds of depression (adjusted OR = 0.26, 95% CI: 0.09–0.76, *p* = 0.014) and, similarly to the overall DASS-42 score, residents of urban areas presented with lower odds of depression versus those residing in rural/semiurban areas (adjusted OR = 0.19, 95% CI: 0.06–0.63, *p* = 0.007).

Regarding anxiety in the DC cohort, once again, residents of urban areas presented with lower odds of anxiety (unadjusted OR = 0.28, 95% CI: 0.09–0.87, *p* = 0.028); no multivariate analysis was undertaken, as the rural/urban status was the only variable associated with anxiety levels.

Concerning stress levels, at the multivariate analysis, females presented with lower odds of stress (adjusted OR = 0.33, 95% CI: 0.11–0.99, *p* = 0.048) and residents of urban areas presented with lower odds of stress versus those residing in rural/semiurban areas (adjusted OR = 0.27, 95% CI: 0.08–0.90, *p* = 0.033). On the other hand, the association between secondary/university education and lower stress levels (unadjusted OR = 0.35, 95% CI: 0.13–0.97, *p* = 0.044) was no longer significant in the multivariate analysis (*p* = 0.170).

## 4. Discussion

From an oncology nursing perspective, the observed differences in psychological distress between day care and long-term hospitalized patients highlight the need for differentiated, context-sensitive nursing approaches across care settings. The results of this study showed a distinct pattern of associations in the LONG and in the DC cohort, highlighting the different social, health system-related, and clinical conditions that patients encountered in the two cohorts.

Female patients exhibited higher levels of psychological distress, depression, stress, and anxiety compared to male patients in the LONG cohort. Existing literature has indicated that gender differences may influence the levels of preoperative anxiety and stress experienced by patients; females often report higher anxiety levels compared to their male counterparts, potentially due to differing coping mechanisms, socialization patterns, and responses to medical settings [9,10]. Scott et al. found that female patients exhibited significantly greater preoperative anxiety, which can be attributed to a heightened fear of surgical interventions [11]. Conversely, other studies have presented contrasting findings, indicating that men may express their stress differently, which can also result in significant psychological distress that is often overlooked [12]. Interestingly, in contrast with the LONG cohort, women presented with lower odds of depression and stress versus males in our DC cohort. Overall, the aforementioned differential patterns in the LONG and DC cohorts highlight the complexities surrounding gender and preoperative anxiety, prompting a need for tailored approaches to managing patient care [13].

Notably, in the LONG cohort, all male participants scored below the DASS-42 distress threshold, a pattern that requires cautious interpretation. Although this finding may reflect a genuine gender-related difference in preoperative psychological responses, the possibility of reporting bias cannot be excluded. Self-reported measures of psychological distress may be influenced by gender norms and sociocultural expectations, potentially affecting the way male patients express or acknowledge emotional symptoms. Therefore, this result should be interpreted within the broader sociocultural context and warrants further investigation in future studies using complementary assessment approaches.

Patients treated at DC were primarily from urban areas (*p* = 0.002), namely the metropolitan area of Athens, where the studied centers are located. Patients from the Athens metropolitan area might have felt safer having surgery in DC, having immediate access to the hospital after discharge to address any possible complications and to receive follow-up care. On the other hand, for patients living in the distant semiurban/rural areas, the LONG option seemed preferable, as patients might consider their transport a problem, especially after discharge; in a DC scenario, the return of rural patients to their remote residence on the same day of the operation would represent a challenge. Indeed, in our DC cohort, residents of urban areas presented with lower odds of psychological distress (depression, anxiety and stress) versus the rural/semiurban ones; the inverse pattern arose in the LONG cohort, where rural/semiurban patients were less affected than those living in the fast-paced metropolis. The DC and LONG cohorts represent structurally different clinical pathways by design. Therefore, differences observed between them should be interpreted as reflections of distinct care environments rather than effects isolated from clinical case-mix. The study deliberately examines these pathways as they naturally occur in routine oncology practice.

Oncology nurses are often the first health care professionals to detect heightened psychological distress in surgical patients, underscoring their pivotal role in systematic distress screening and timely psychosocial support. The findings suggest that nurse-led preoperative interventions, including patient-centered communication and anxiety-reduction strategies, could be tailored according to the type and duration of hospitalization in oncology care.

The literature has well established the challenges of rural residence in terms of increased anxiety levels and a lack of access to mental health resources, in turn affecting surgical preparation and recovery [14,15]. On the other hand, patients from urban settings may sometimes face environmental stressors that could exacerbate anxiety and depression, such as the fast-paced lifestyle [5,6], financial pressures, increased competition for medical resources, noise pollution, crowded health care facilities [16], and population density [17,18]. Regarding the challenges imposed by rural/urban status, recent studies have shown that telehealth interventions could effectively cater to patients’ informational needs and psychological concerns, ultimately leading to decreased anxiety prior to surgery [19] and enhancing patient readiness for surgical procedures; hybrid models combining traditional in-person interactions with telemedicine approaches have also been useful [20]. Nevertheless, telehealth interventions that could potentially mitigate psychological distress in rural patients were not available in our setting.

Regarding surgical severity in the LONG cohort, a difficult-to-interpret association emerged, with major and moderate surgery presenting with lower psychological distress; it would be tempting to hypothesize that patients undergoing milder surgeries perceived the long-term hospitalization facility (LONG cohort) as a rather stressful context and might have preferred shorter care, closer to DC. No associations with the severity of surgery arose in the DC cohort, which in any case did not include any major surgical procedures. This counterintuitive finding underscores the complexity of psychological adaptation in oncological surgery and suggests that perceived seriousness of illness and preoperative preparation processes may influence distress levels more strongly than surgical magnitude per se. Nevertheless, this interpretation should be approached with caution given the observational design of the study.

Comparing the psychological distress levels in the two cohorts (DC vs. LONG), no significant differences were identified between them, except for stress levels, which were more pronounced in the DC cohort than in the LONG cohort (*p* = 0.05). As DC surgery becomes more common due to its cost-effectiveness and efficiency, understanding the psychological landscape of patients in this context is crucial [21]. Research has shown that preoperative stress remains pronounced in individuals undergoing elective procedures [22]. Moreover, although the WT was substantially longer in the DC cohort (*p* = 0.002), the stratified analyses within each cohort did not reveal any independent association of waiting time (WT) (above vs. below median WT in each cohort) with psychological distress. Overall, the lack of substantial differences in total psychological distress between the DC and LONG cohorts suggests that the hospitalization model per se may not constitute the primary determinant of preoperative distress. Given the non-randomized allocation and the exclusive performance of major surgeries in the LONG cohort, comparisons between hospitalization models should be interpreted as descriptive rather than causal. The observed differences may reflect underlying clinical case-mix rather than the structural characteristics of the hospitalization model itself.

Several limitations should be acknowledged. First, the complete separation observed for gender in the LONG cohort likely reflects sample characteristics within this specific clinical pathway rather than a universalizable pattern. Community support structures [23] and cultural frameworks of patients’ residential areas [11] may have influenced the findings related to rural/urban status; however, such contextual variables were not formally assessed within the study protocol. In addition, detailed clinical characteristics, including cancer type, specific surgical procedures, comorbidities, and prior psychiatric history, were not systematically recorded, although these factors may substantially affect psychological distress and postoperative experience [24]. Second, the study was conducted in two cohorts within a single oncology hospital in Greece, which may limit generalizability to other health care systems and cultural settings. A key methodological limitation concerns the clinical imbalance between the DC and LONG cohorts, particularly regarding surgical severity, as major procedures were exclusively performed in the LONG cohort. This restricts internal comparability and introduces potential confounding, precluding causal attribution of psychological distress to the hospitalization model alone. From a statistical perspective, the dichotomization of continuous variables such as age and waiting time may have reduced analytical precision. Furthermore, complete separation in certain regression models (e.g., gender in the LONG cohort) suggests possible model instability. This statistical phenomenon resulted in unstable parameter estimates with inflated odds ratios and wide confidence intervals, limiting the precision and reliability of specific associations (particularly gender effects in the LONG cohort). These findings should therefore be interpreted as exploratory rather than confirmatory. The extremely low odds ratios observed for higher surgical severity, along with the absence of adjustment for multiple comparisons, warrant cautious interpretation. Additionally, no formal correction for multiple comparisons was applied despite the number of regression analyses performed. Therefore, the possibility of a type I error cannot be excluded, and statistically significant findings should be interpreted with appropriate caution. Finally, the relatively modest sample size may have constrained statistical power.

These results reinforce the critical contribution of oncology nursing to the holistic management of surgical patients, particularly in addressing psychological distress within evolving oncology care environments. Future research should explore the effectiveness of structured, nurse-led psychosocial interventions aimed at reducing psychological distress in surgical oncology patients across different hospitalization models.

Overall, the findings suggest that preoperative psychological distress was not substantially differentiated by hospitalization model, but was more strongly associated with gender, place of residence, and severity of surgery. These results highlight the importance of individualized psychosocial assessment rather than model-based assumptions. For oncology nursing practice, structured preoperative distress screening that accounts for sociodemographic and clinical characteristics may be particularly beneficial across both day care and long-term hospitalization settings.

## 5. Conclusions

Gender, rural/urban residence, and surgical severity were associated with preoperative distress; however, given the observational design and structural group imbalance, these associations should not be interpreted as causal determinants. Studies assessing gender-specific dynamics and mixed-methods studies could provide deeper insights into patients’ experiences and the evaluation of factors affecting preoperative psychological distress.

## Figures and Tables

**Table 1:** Demographic and clinical characteristics of patients in the Day Care (DC) and Long-Term Hospitalization (LONG) cohorts.

Mean (SD)	DC *n* = 88	LONG *n* = 88	*p*-Value
Age	56.2 (16.6)	57.9 (14.0)	t = −0.719. *p* = 0.473
Gender			Chi^2^ = 0.25. df = 1. *p* = 0.613
Male	23 (26.1)	26 (29.5)	
Female	65 (73.9)	62 (70.5)	
Education level			Chi^2^ = 5.05. df = 3. *p* = 0.168
No education	0 (0)	1 (0.6)	
Primary education	45 (51.1)	55 (62.5)	
Secondary education	37 (42)	24 (27.3)	
University degree	6 (6.8)	8 (9.1)	
Marital Status			Chi^2^ = 2.51. df = 3. *p* = 0.472
Single	13 (14.8)	9 (10.2)	
Married	53 (60.2)	48 (54.5)	
Divorced	11 (12.5)	15 (17)	
Widowed	11 (12.5)	16 (18.2)	
Residence			Chi^2^ = 12.98. df = 2. *p* = 0.002
Semiurban	7 (8)	18 (20.5)	
Urban	72 (81.8)	50 (56.8)	
Rural	9 (10.8)	20 (22.7)	
Professional status			Chi^2^ = 7.79. df = 6. *p* = 0.253
Unemployed	19 (21.6)	21 (23.9)	
Household	4 (4.5)	7 (8)	
College student	1 (1.1)	2 (2.3)	
Public employee	13 (14.8)	13 (14.8)	
Private employee	31 (35.2)	17 (19.3)	
Freelancer	9 (10.2)	17 (19.3)	
Retired	11 (12.5)	11 (12.5)	
Severity Surgery Grading			Chi^2^ = 93.65. df = 2. *p* < 0.001
Mild	51 (58)	5 (5.7)	
Moderate	37 (42)	29 (33)	
Major	0 (0)	54 (61.3)	
First time for operation			Chi^2^ = 3.51. df = 1. *p* = 0.088
No	70 (79.5)	59 (67)	
Yes	18 (20.5)	29 (33)	
Waiting Time (months)	1.32 (1.64)	0.69 (0.66)	U = 2884. *p* = 0.002
DASS Total	25.5 (25.0)	20.5 (23.6)	U = 3283.5. *p* = 0.081
DASS Depression	7.6 (9.3)	6.4 (8.5)	U = 3715. *p* = 0.639
DASS Anxiety	7.2 (7.8)	5.8 (7.5)	U = 3384. *p* = 0.146
DASS Stress	10.8 (9.5)	8.4 (9.0)	U = 3224.5. *p* = 0.05

**Table 2:** Results of univariate and multivariate logistic regression analyses for the DASS-42 total score in the LONG and DC cohorts.

		LONG Cohort				DC Cohort			
		Unadjusted OR (95% CI)	*p*	Adjusted OR (95%CI)	*p*	Unadjusted OR (95% CI)	*p*	Adjusted OR (95%CI)	*p*-Value
Overall DASS score (mild/moderate/high/very high vs. normal)									
Gender	Female vs. male	Not estimable because all males scored in DASS total < 33	*p* < 0.001 (Fisher’s exact test)			0.39 (0.14–1.07)	0.067		
Age	≥median vs. <median	0.69 (0.26–1.84)	0.454			1.23 (0.49–3.13)	0.659		
Education	Secondary/university vs. primary/none	0.33 (0.10–1.08)	0.067			0.48 (0.18–1.25)	0.132		
Marital status	Married vs. single/divorced/widowed	0.89 (0.33–2.38)	0.819			0.78 (0.31–2.00)	0.610		
Place of residence	Urban vs. rural/semiurban	3.11 (1.02–9.45)	0.046	4.66 (1.22–17.81)	0.024	0.16 (0.05–0.50)	0.002		
Professional status	In work/studying vs. unemployed/household/retired	1.08 (0.40–2.91)	0.877			2.49 (0.85–7.33)	0.097		
WT (months)	≥median vs. <median	0.49 (0.18–1.36)	0.170			1.09 (0.43–2.76)	0.863		
Severity of surgery	Major vs. mild	0.04 (0.004–0.44)	0.008	0.02 (0.002–0.3)	0.004				
	Moderate vs. mild	0.11 (0.01–1.15)	0.66	0.06 (0.01–0.76)	0.030	0.70 (0.27–1.83)	0.470		
First operation	Yes vs. no	0.77 (0.26–2.24)	0.625			1.17 (0.45–3.05)	0.749		

**Table 3:** Results of univariate and multivariate logistic regression analyses for the DASS-42 depression, anxiety, and stress subscales in the LONG and DC cohorts.

		LONG Cohort				DC Cohort			
		Unadjusted OR (95% CI)	*p*	Adjusted OR (95%CI)	*p*	Unadjusted OR (95% CI)	*p*	Adjusted OR (95%CI)	*p*-Value
DASS depression score (mild/moderate/high/very high vs. normal)									
Gender	Female vs. male	Not estimable because all males scored in DASS depression < 9	*p* = 0.002 (Fisher’s exact test)			0.30 (0.11–0.82)	0.019	0.26 (0.09–0.76)	0.014
Age	≥median vs. <median	0.86 (0.30–2.49)	0.787			1.155 (0.60–3.97)	0.362		
Education	Secondary/university vs. primary/none	0.31 (0.08–1.18)	0.086			0.61 (0.24–1.55)	0.297		
Marital status	Married vs. single/divorced/widowed	1.24 (0.42–3.63)	0.694			0.78 (0.31–2.00)	0.610		
Place of residence	Urban vs. rural/semiurban	2.97 (0.89–10.05)	0.077			0.22 (0.07–0.69)	0.009	0.19 (0.06–0.63)	0.007
Professional status	In work/studying vs. unemployed/household/retired	0.65 (0.22–1.88)	0.427			1.17 (0.45–3.05)	0.749		
WT (months)	≥median vs. <median	0.56 (0.19–1.68)	0.300			0.87 (0.34–2.19)	0.763		
Severity of surgery	Major vs. mild	0.03 (0.002–0.27)	0.002						
	Moderate vs. mild	0.10 (0.01–0.99)	0.049			0.70 (0.27–1.83)	0.470		
First operation	Yes vs. no	0.22 (0.05–1.03)	0.054			1.14 (0.62–5.49)	0.273		
DASS anxiety score (mild/moderate/high/very high vs. normal)									
Gender	Female vs. male	16.89 (2.15–132.88)	0.007	20.78 (2.10–205.38)	0.009	0.56 (0.21–1.47)	0.237		
Age	≥median vs. <median	0.80 (0.32–2.01)	0.641			0.65 (0.27–1.54)	0.322		
Education	Secondary/university vs. primary/none	0.42 (0.15–1.18)	0.099			0.80 (0.34–1.91)	0.620		
Marital status	Married vs. single/divorced/widowed	1.20 (0.48–3.02)	0.702			1.03 (0.42–2.48)	0.955		
Place of residence	Urban vs. rural/semiurban	1.66 (0.64–4.29)	0.296			0.28 (0.09–0.87)	0.028		
Professional status	In work/studying vs. unemployed/household/retired	1.12 (0.45–2.83)	0.806			0.78 (0.32–1.87)	0.572		
WT (months)	≥median vs. <median	0.60 (0.23–1.59)	0.308			0.56 (0.23–1.33)	0.186		
Severity of surgery	Major vs. mild	0.07 (0.01–0.70)	0.023	0.05 (0.003–0.92)	0.044				
	Moderate vs. mild	0.13 (0.01–1.34)	0.087	0.06 (0.003–1.09)	0.057	0.84 (0.35–2.02)	0.696		
First operation	Yes vs. no	0.67 (0.24–1.84)	0.437			1.92 (0.67–5.46)	0.233		
DASS stress score (mild/moderate/high/very high vs. normal)									
Gender	Female vs. male	Not estimable because all males scored in DASS stress <14	*p* = 0.001 (Fisher’s exact test)			0.33 (0.12–0.91)	0.031	0.33 (0.11–0.99)	0.048
Age	≥median vs. <median	0.59 (0.22–1.63)	0.312			1.26 (0.48–3.28)	0.635		
Education	Secondary/university vs. primary/none	0.36 (0.11–1.18)	0.092			0.35 (0.13–0.97)	0.044	0.45 (0.16–1.38)	0.170
Marital status	Married vs. single/divorced/widowed	0.79 (0.29–2.14)	0.643			1.04 (0.39–2.74)	0.942		
Place of residence	Urban vs. rural/semiurban	4.00 (1.21–13.21)	0.023	7.19 (1.52–34.07)	0.013	0.26 (0.08–0.82)	0.021	0.27 (0.08–0.90)	0.033
Professional status	In work/studying vs. unemployed/household/retired	1.26 (0.46–3.46)	0.659			1.25 (0.46–3.37)	0.659		
WT (months)	≥median vs. <median	0.58 (0.21–1.65)	0.307			1.42 (0.54–3.74)	0.479		
Severity of surgery	Major vs. mild	0.04 (0.004–0.44)	0.008	0.02 (0.001–0.26)	0.003				
	Moderate vs. mild	0.10 (0.01–0.99)	0.049	0.04 (0.003–0.55)	0.017	0.66 (0.25–1.78)	0.413		
First operation	Yes vs. no	0.61 (0.20–1.89)	0.392			2.15 (0.72–6.45)	0.173		

## Data Availability

The raw data supporting the conclusions of this article will be made available by the authors on request.
